# Molecular insight into hypervirulent *Klebsiella pneumoniae* from North India

**DOI:** 10.6026/973206300220009

**Published:** 2026-01-31

**Authors:** Rashmi, Vimala Venkatesh, Sheetal Verma, Upma Singh, Mohit

**Affiliations:** 1 Department of Microbiology, Faculty of Medicine, King George's Medical University, Lucknow, India

**Keywords:** Hypervirulent *Klebsiella pneumoniae*, virulence biomarkers, hypermucoviscosity, siderophores, mucoid phenotype, classical *Klebsiella pneumoniae*

## Abstract

Hypervirulent *Klebsiella pneumoniae* (hvKp) is an emerging pathotype capable of causing tissue-invasive, community- acquired infections in healthy individuals across all age groups. This strain is characterized by hypermucoviscosity and the presence of specific virulence biomarker genes, which contribute to its enhanced pathogenicity compared to classical *Klebsiella pneumoniae* (cKp). For better patient care and epidemiological research, we need a reliable test to find hvKp strains. Therefore, it is of interest to differentiate between cKp and hvKp strains by assessing hypermucoviscosity using the string test (≥5 mm string length) and detecting virulence biomarkers via PCR assay. Among 100 isolates, 37 (37%) tested positive for the string test and molecular analysis identified 28 (28%) as hvKp. Of the 28% hvKp strains, 13 (46.4%) exhibited hypermucoviscosity and hypervirulence. All hvKp strains 28 (100%) carried at least one siderophore biosynthesis gene (*iucA*, *iucB* and *iroB*), 23 (82.1%) were positive for mucoid phenotype genes (*rmpA*, *rmpA*2), and 11 (39.2%) harbored the *peg344* gene encoding a putative metabolite transporter. Notably, 5 (17.9%) hvKp strains possessed all six major virulence genes (*rmpA*, *rmpA*2, *iucA*, *iucB*, *iroB* and *peg344*). Thus, we show that virulence biomarkers such as siderophore biosynthesis and mucoid phenotype genes are critical for identifying hvKp strains. The integration of hypermucoviscosity and hypervirulence underscores the necessity for focused research and heightened clinical vigilance to combat this emerging threat.

## Background:

*Klebsiella pneumoniae* is a Gram-negative opportunistic pathogen responsible for healthcare-associated infections like sepsis, Urinary Tract Infections and pneumonia [1]. A critical trait of *K. pneumoniae* that has enabled its ongoing evolution is the ability to acquire new genetic material. As a result, two pathotypes termed classical *K. pneumoniae* (cKp) and hypervirulent *K. pneumoniae* (hvKp) are presently active in circulation [2]. The cKp usually causes infections in hospitals in individuals who already have other health problems, have weakened immune systems, or have encountered physical barriers such surgical incisions or burns [3]. In contrast, hvKp strains pose a novel and more significant threat. In contrast with standard strains, these variants possess the ability to infiltrate and harm host tissues in individuals who are otherwise healthy, frequently resulting in severe infections like liver abscesses, meningitis and necrotising fasciitis. Their capacity to bypass the immune system and proliferate rapidly has drawn significant worldwide concern. This pathotype possesses the ability to obtain a diverse array of genetic elements that confer multidrug resistance to multiple classes of antibiotics [3- 4]. Initial cases of hvKp infections were recorded in the Asia Pacific Rim and have since spread globally, including Australia, the Americas, Europe and Africa [5]. In healthy individuals, hvKp can lead to severe infections like endophthalmitis, meningitis, hepatic abscesses and bacteremia. While hvKp strains are typically antibiotic-susceptible, they show high virulence [6-7]. Studies indicate that hvKp strains, evolving from cKp, show hypermucoviscous traits, evidenced by a positive "string test" where a colony stretches 5 mm or more. However, it's unclear if all hvKp strains possess these properties [8- 9].

The hypervirulent pathotype of *K. pneumoniae* is characterized by large virulent plasmids, such as pLVPK-like 220-kb pK2044, 219-kb pLVPK and 230-kb pRJA166b, or by chromosomal islands [10, 11- 12]. The virulence plasmids were characterized using whole genome sequencing (WGS), revealing the presence of hypervirulent genes such as *iucA*BCD, iutA (aerobactin), *iroB*CDN (salmochelin), *rmpA*, *rmpA*2 and peg-344. These genetic markers indicate hypervirulence [13- 14]. The regulator of mucoid phenotype genes boosts bacterial capsule production, leading to hypermucoviscous colony morphology, as shown in several studies [15, 16, 17-18]. These capsules help the bacterium resist the host immune response by impeding phagocytosis, complement-mediated killing and opsonization. Hypermucoviscosity also limits DNA movement, reducing gene exchange [7]. Siderophores are molecules secreted by bacteria to scavenge iron. *K. pneumoniae* isolates may produce up to four siderophores. Biosynthesis genes for salmochelin (*iroB* CDN) and aerobactin (*iucA*BCD, iutA) are mostly harbored by hvKp strains. Classical pathotype predominantly produces only enterobactin (*entB*). Biosynthesis genes of yersiniabactin (*ybt*) are linked to genotoxin colibactin (*clb*) genes in some hvKp and cKp [13, 19]. The hvKp-specific metabolite transporter (*peg344*), located on the virulence plasmid, enhances virulence in pulmonary infections and facilitates multi-site infections [17]. Therefore, it is of interest to report that the specific genes or their phenotypes could serve as useful indicators for identifying hypervirulent *K. pneumoniae* (hvKp) strains. So, we looked at a few genotypic and phenotypic biomarkers to determine how effectively they could distinguish the difference between putative hvKp and cKp strains taken from several kinds of clinical samples.

## Materials and Methods:

## Location:

This study was conducted in the laboratory of Bacteriology of the Department of Microbiology at King George's Medical University, Lucknow and Uttar Pradesh, India.

## Collection, Isolation and Identification:

Clinical bacterial isolates were obtained from various specimens, including blood, urine, pus, cerebrospinal fluid (CSF), respiratory samples and body fluids. All samples were cultured on MacConkey and blood agar and incubated at 37°C. Identification was based on cultural characteristics, morphology, standard biochemical tests and confirmed using the MALDI-TOF MS system. All *K. pneumoniae* isolates were stored in 50% glycerol stock at -20°C. A total of 100 non-duplicate isolates were revived by subculturing on MacConkey agar for further analysis.

## String test:

The hypermucoviscous phenotype of *Klebsiella pneumoniae* isolates was assessed using the string test as described in previous studies [6, 20-21]. The test is considered positive when a sterile inoculating loop can produce a viscous string ≥5 mm in length by stretching a single bacterial colony on blood agar after incubation at 37°C for 24 hours. This method serves as a key phenotypic indicator of hypermucoviscosity.

## DNA extraction:

Genomic DNA was extracted from bacterial isolates using the conventional boiling method. Briefly, 2-3 fresh bacterial colonies were suspended in 200 µL of nuclease-free water (Thermo Scientific). The suspension was lysed by heating at 95°C for 20 minutes in a dry water bath. Following lysis, cellular debris was removed by centrifugation at 12,000 rpm for 2-5 minutes and the resulting supernatant was used as a DNA template for PCR (Polymerase chain reaction) amplification.

## Identification of hypervirulent *Klebsiella pneumoniae* (hvKp):

Hypervirulent *Klebsiella pneumoniae* (hvKp) strains were identified using PCR amplification of specific virulence biomarker genes, including plasmid-borne regulators of the mucoid phenotype (*rmpA*, *rmpA*2), aerobactin siderophore biosynthesis genes (*iucA*, *iucB*), salmochelin siderophore biosynthesis gene (*iroB*) and the gene encoding a putative metabolite transporter (*peg344*) [14, 22, 23, 24, 25-26]. The PCR conditions, specific primers and expected product sizes for each target gene are detailed in Table 1. These virulence biomarkers provide robust molecular evidence to differentiate hvKp from cKp strains.

## Statistical analysis:

All data were recorded in Microsoft Excel and analyzed using MedCalc statistical software (MedCalc Software Ltd., Version 23.0.9). The "Comparison of Means Calculator" was utilized to evaluate differences in data sets (https://www.medcalc.org/calc/comparison_of_means.php). A P-value of less than 0.05 was considered statistically significant.

## Results:

**Figure 1:**
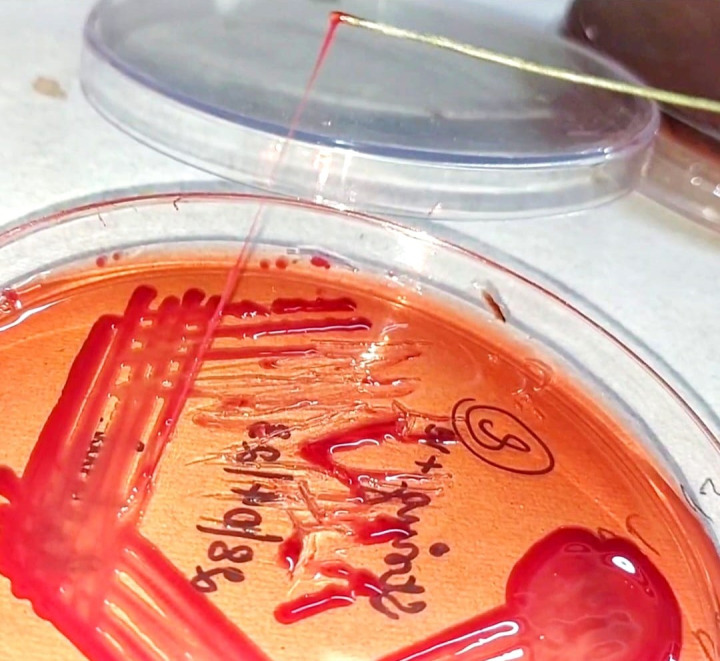
String test depicting hypermucoviscosity in a *Klebsiella pneumoniae* isolate.

**Figure 2:**
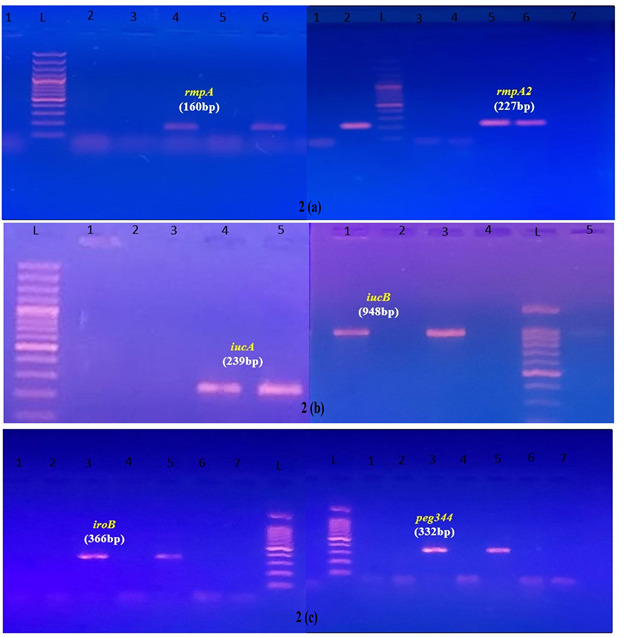
Gel Electropherogram images of PCR products of virulence biomarker genes in *Klebsiella pneumoniae* isolates. (a) Gel Electrophoretogram of PCR product of Mucoid phenotype of the virulence gene. L: DNA ladder, no. 4 and 6: *rmpA* gene (160bp); no. 2, 5 and 6: *rmpA*2 gene (227bp). (b) Gel Electrophoretogram of PCR product of siderophore biosynthesis genes (Aerobactin). L: DNA ladder, no. 4 and 5: *iucA* gene (239bp); no. 1 and 3: *iucB* gene (948bp). (c) Gel Electrophoretogram of PCR product of siderophore biosynthesis gene (Salmochelin) and putative metabolite transporter gene. L: DNA ladder, no. 3 and 5: *iroB* gene (366bp); no. 3 and 5 peg344 gene (332bp).

**Figure 3:**
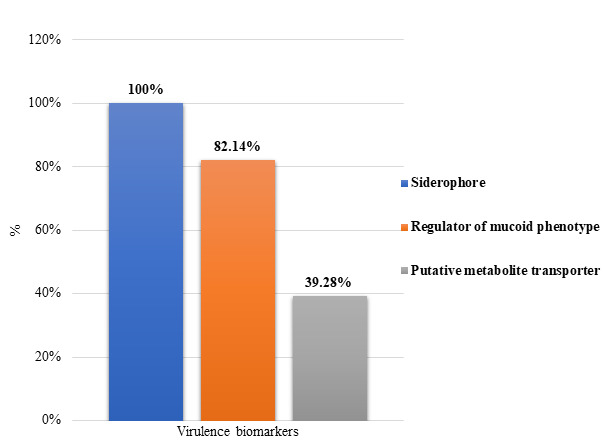
The combined distribution of virulence biomarker genes.

**Figure 4:**
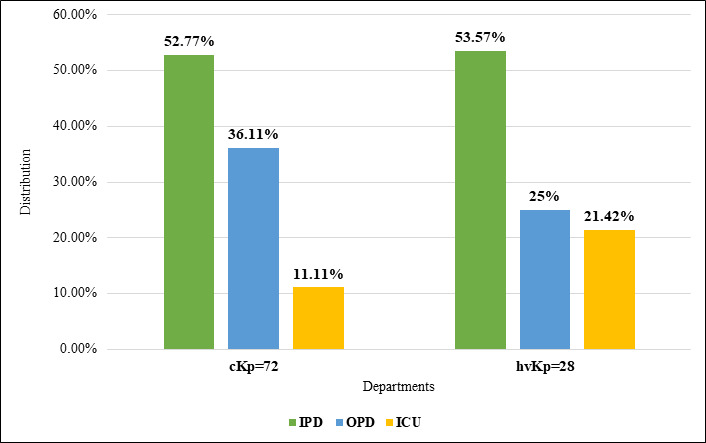
Distribution of cKp and hvKp isolates across different wards and departments. (GW/IPD: General wards/Inpatient department; OPD: Outpatient department; ICU: Intensive care unit)

Out of the 100 *Klebsiella pneumoniae* isolates evaluated, 37 (37%) were identified as hypermucoviscous based on a positive string test (≥5mm) shown in Figure 1. PCR amplification revealed that a notable proportion of *K. pneumoniae* isolates exhibited virulence biomarker genes (*rmpA*, *rmpA*2, *iucA* and *iucB*). Out of the 100 isolates studied, 28 (28%) were identified as hvKp strains due to the presence of specific virulence genes (Table 2). These results were validated in Figure 2a, b, c by the Gel Electropherogram analysis of the PCR-obtained products from the isolates. The remaining 72 (72%) were categorized as classical *K. pneumoniae* (cKp). Interestingly, 13 (46.42%) of the 28 hvKp strains were positive for the string test, confirming their hypermucoviscosity and hypervirulence. Figure 3 summarizes the overall prevalence of virulence biomarker genes in *K. pneumoniae* isolates. PCR data analysis revealed that the combination of the *rmpA*, *rmpA*2 and *iucA* genes was the most prevalent among the hvKp strains, occurring in 19 (67.85%). Additionally, 5 hvKp strains, representing 17.85%, exhibited all six virulence biomarker genes: *rmpA*, *rmpA*2, *iucA*, *iucB*, *iroB* and *peg344* (Table 3). 100 *Klebsiella pneumoniae* isolates were obtained from various clinical specimens, including blood, urine, exudates, cerebrospinal fluid (CSF), respiratory samples and body fluids. The hvKp strains were most commonly isolated from exudate samples 8 (28.57%) and blood samples 7 (25%), as detailed in Table 4. Analysis of the distribution of *K. pneumoniae* pathotypes across different wards and departments revealed a balanced representation of cKp (72%) and hvKp (28%). Both cKp 38 (52.77%) and hvKp 15 (53.57%) were predominantly isolated from patients in general inpatient departments (IPD). Notably, the hvKp strains were more frequently associated with patients in intensive care units (ICU) 6 (21.42%) compared to cKp strains 8 (11.11%). This discrepancy suggests a potential link between hvKp infections and severe cases necessitating critical care or may indicate superior diagnostic capabilities in ICU settings (Figure 4).

## Discussion:

Hypervirulent *Klebsiella pneumoniae* (hvKp) is defined clinically by its ability to cause tissue-invasive, community-acquired infections in otherwise healthy individuals, excluding those with comorbidities, immunosuppression, or healthcare- associated infections [4]. Hypervirulence and hypermucoviscosity are related but distinct phenotypes, often coexisting but not synonymous. Identification of the genetic determinants of hypervirulence is therefore crucial. A study at Beijing Chao-Yang Hospital (2008-2012) found 31.4% (22/70) of isolates were hvKp based on positive string tests [30]. While hypermucoviscosity often occurs in hvKp, classical *K. pneumoniae* (cKp) can also display it due to capsular locus mutations [31]. Siderophore genes *iucA* and *iroB* show high predictive accuracy for hypervirulence (96% and 97%, respectively) [32- 33]. Other important contributors include mucoid regulators and genes *rmpA*, *rmpA*2, peg-344, *iucA* and *iroB*, with *iucA* (aerobactin siderophore) being a key virulence factor that enhances iron acquisition and bacterial survival [14, 29, 34-35]. The prevalence of hvKp varies regionally. Yang *et al.* (2020) reported 52.2% (59/113) hvKp, Egypt ICU patients showed 6.15% and Eastern China studies found *iucA*, *rmpA* and *iroB* in 56.8%, 43.2% and 40.9% of isolates, respectively [36, 37-38]. A multicenter study in China (2013) observed 37.8% hvKp, ranging from 73.9% in Wuhan to 8.3% in Zhejiang [39]. Sanikhani R *et al.* found 45 isolates had only *iucA* gene, but only 3 isolates had multiple virulence genes [27]. A meta-analysis study also indicated that the prevalence of hvKp strains was 33.0%, primarily based on research conducted in Asia, predominantly in China [40]. Recent studies have consistently documented the increasing prevalence of multidrug-resistant and hypervirulent strains of *K. pneumoniae* [41]. The increasing prevalence of antibiotic resistance is constraining available treatment options for diseases caused by drug-resistant bacterial strains. Worldwide, Greece demonstrates the highest documented prevalence of carbapenem resistance, at 68%. This is succeeded by India and the Eastern Mediterranean region, both of which report rates of 54% [42]. These variations may reflect differences in methodology, clinical practices, or strain heterogeneity. In our study of 100 *K. pneumoniae* isolates, we used molecular markers *rmpA*, *rmpA*2 and *iucA* for hvKp detection. Results showed 28% were hvKp by molecular markers, while 37% were hypermucoviscous by the string test. Among hvKp strains, *iucA* was present in 85.7%, *rmpA* in 82.1% and *rmpA*2 in 78.6%, highlighting their roles in virulence and capsular polysaccharide (CPS) synthesis. Hypervirulence and hypermucoviscosity overlapped in 46.4% of strains. Plasmid-borne genes *iroB* and peg-344 were less common (42.9% and 39.3%) and 17.9% carried all six virulence markers (*rmpA*, *rmpA*2, *iucA*, *iucB*, *iroB*, peg-344), representing highly pathogenic strains. This study demonstrates that using multiple molecular markers improves hvKp detection over phenotypic methods. *iucA* remains a critical biomarker across regions. Our findings reveal variability in virulence gene profiles and highlight strains with multi-gene virulence potential, particularly in ICU settings, emphasizing the need for strict infection control. Future research should examine clinical outcomes, additional genetic factors and the role of less-studied genes like *iroB* and peg-344 in hypervirulence.

## Conclusion:

The presence of *iucA*, *rmpA* and *rmpA*2 genes can serve as significant molecular markers for identifying hvKp isolates, providing reliable indicators for distinguishing hypervirulent strains. Conversely, the lower prevalence of *iroB* and peg-344 suggests that while they contribute to the pathogenicity of certain strains, their presence is less consistent compared to the core markers. These findings underscore the genetic and phenotypic heterogeneity of *Klebsiella pneumoniae* infections, emphasizing the clinical importance of hvKp as an emerging threat capable of causing severe, invasive infections even in healthy individuals. The identification of critical virulence factors such as *rmpA*, *rmpA*2, *iucA* and *iucB* underscores the necessity for rapid and precise diagnostic tools to differentiate hvKp from classical strains. This distinction is crucial for implementing appropriate therapeutic interventions and enhancing infection control measures. The study also reveals the broader epidemiological context, noting the higher prevalence of hvKp in severe cases, particularly among ICU patients, which necessitates vigilant surveillance and tailored strategies to mitigate the risks associated with these highly virulent strains in healthcare settings. By shedding light on the molecular and phenotypic characteristics of hvKp, this research provides a robust foundation for understanding its clinical significance and epidemiological distribution. It underscores the pressing need for global and regional efforts to monitor and manage hvKp infections effectively. Future investigations should focus on exploring additional genetic markers, resistance mechanisms and host-pathogen interactions to further delineate the pathogenic landscape of *Klebsiella pneumoniae*. Thus, it is required to develop innovative and targeted strategies to combat the rising threat posed by hypervirulent *Klebsiella pneumoniae* globally.

## Author contribution:

VV designed the research, supervised it, provided resources and reviewed and edited the manuscript. R conducted all experiments and data analysis and wrote manuscripts (drafting, reviewing and editing). SV monitored experiments. US and M manuscript review and editing assistance.

## Funding:

Ms. Rashmi is supported by the Senior Research Fellowship (SRF) from the Council of Scientific and Industrial Research, Government of India. The funders did not participate in the study design, data collection and analysis, decision-making regarding publication, or manuscript preparation.

## Figures and Tables

**Table 1:** Primers and product sizes for virulence biomarker genes

**S.N.**	**Gene Primer**	**Primer sequence (5'→3') (Forward & Reverse)**	**Amplicon size (bp)**	**Annealing temperature**	**References**
1	*rmpA*	F: ATGTGGCTTGACGTTTCGGGGG	160bp	60°C	[27]
		R: GCCGTGGATAATGGTTTACAATTCGGC			
2	*rmpA*2	F: GGATGTGGCTTGACATTTCGGGGG	227bp	60°C	
		R: TTCATGGATGCCCTCCCTCCTG			
3	*iucA*	F: AATCAATGGCTATTCCCGCTG	239bp	62°C	[28]
		R: CGCTTCACTTCTTTCACTGACAGG			
4	*iucB*	F: ATGTCTAAGGCAAACATCGT	948bp	49°C	[29]
		R: TTACAGACCGACCTCCGTGA			
5	*iroB*	F: GTGTTGGATTCCGCCAGTGA	366bp	61°C	[28]
		R: TTCCGCCGCTACCTCTTCA			
6	*peg344*	F: GCGGGAAAGGACAGAAAGCCAGTG	332bp	56°C	
		R: GAGGGAAGATGAGAAATACGAGC			

**Table 2:** Distribution of virulence biomarkers among hvKp strains

**S.N.**	**Virulence biomarkers**	**Target gene**	**Total distribution n=28 (%)**	
1	Siderophore biosynthesis			28 (100%)
	Aerobactin	*iucA*	24 (85.71%)	
		*iucB*	16 (57.14%)	
	Salmochelin	*iroB*	12 (42.85%)	
2	Mucoid phenotype	*rmpA*	23 (82.14%)	23 (82.14%)
		*rmpA*2	22 (78.57%)	
3	Putative metabolite transporter protein	*peg344*	11(39.28%)	23 (82.14%)

**Table 3:** Combinations of virulence biomarker genes in hvKp isolates

**S.No.**	**Combinations**	**Total hvKp strains n=28 (%)**
1	*rmpA*+*rmpA*2+*iucA*	19 (67.85%)
2	*rmpA*+*rmpA*2+*iucA*+*iucB*	12 (42.85%)
3	*rmpA*+*rmpA*2+*iucA*+*iucB*+*iroB*	5 (17.85%)
4	*rmpA*+*rmpA*2+*iucA*+*iucB*+*iroB*+*peg344*	5 (17.85%)

**Table 4:** Distribution of cKp and hvKp isolates across various clinical specimens

**Clinical specimens**	***K. pneumoniae* pathotype n (%)**		**P-value**
Total (n=100)	cKp n=72 (%)	hvKp n=28 (%)	
Blood (20)	13 (18.05)	7 (25.00)	0.1797
Urine (31)	29 (40.27)	2 (7.14)	<0.0001*
Respiratory fluids (18):			
TT (2)	2 (2.77)	-	NA
ET (7)	3 (4.16)	4 (14.28)	0.7055
Sputum (6)	4 (5.55)	2 (7.14)	0.4142
BAL (3)	2 (2.77)	1 (3.57)	0.5637
Exudate (18) (Pus/drain/swab/abscess)	10 (13.88)	8 (28.57)	0.6374
Body fluids (9)	7 (9.72)	2 (7.14)	0.0956
CSF (4)	2 (2.77)	2 (7.14)	NA
TT: Tracheal tube;
ET: Endotracheal tube;
BAL: bronchoalveolar lavage;
CSF: Cerebrospinal fluid;
*: A P value of < 0.05 was
considered to be statistically significant
